# Schwannoma in Digital Nerve: A Rare Case Report

**Published:** 2015-10-24

**Authors:** Jared Troy, Connor Barnes, Andres Gaviria, Wyatt Payne

**Affiliations:** ^a^Division of Plastic Surgery, Department of Surgery, University of South Florida Morsani College of Medicine, Tampa; ^b^Institute for Tissue Regeneration, Repair, and Rehabilitation, Department of Veteran Affairs, Bay Pines VA Health System, Bay Pines, Fla

**Keywords:** schwannoma, neurilemmoma, digital nerve tumor, upper extremity, hand

## DESCRIPTION

A 60-year-old man presented for evaluation of a mobile subcutaneous mass located on the proximal radial aspect of the right-hand small finger. The mass was tender on deep palpation, had a negative Tinel sign, was nonmobile in the axial direction, but was mobile in the volar/dorsal direction.

## QUESTIONS

1. **What is the differential diagnosis of a soft-tissue hand mass?**
2. **How do schwannomas usually present?**
3. **How do schwannomas differ from neurofibromas and malignant peripheral nerve sheath tumor (MPNST)?**
4. **How are schwannomas diagnosed and managed?**

## DISCUSSION

Understanding the differential diagnosis of tumors of the hand is imperative for effective care. A detailed history and physical examination cannot be overemphasized. The differential includes the follow soft-tissue masses: ganglions, epidermal inclusion cysts, foreign-body granulomas, fibromas, tophaceous pseudogout, vascular aneurysms, vascular malformations, giant cell tumors of the tendon sheath, fibroma of the tendon sheath, lipoma, extraosseous chrondroma, leiomyoma, granular cell tumor, MPNST, and, finally, schwannomas. The most common soft-tissue and bone mass in the hand is a ganglion and an enchondroma, respectively.^1^

Schwannomas, also known as neurilemmoma, are benign tumors originating from Schwann cells. Of the benign peripheral nerve tumors, which include schwannomas, neurofibromas, granular cell tumors, neurothekeomas, lipofibromatous hamartomas, or sclerosing perineuriomas, schwannomas are the most common. Schwannomas usually present in the third to fifth decades of life as solitary encapsulated lesions, which are commonly associated with neurofibromatosis type 2; however, incidence is reported as 0.62 per 100,000 population.^2^^-^5 They usually appear as slowly growing subcutaneous masses that are well circumscribed with good lateral mobility and limited axial mobility on palpation. They occur more often in mixed nerves instead of the pure sensory or motor nerve.^2^^-^5 Most are asymptomatic but can become painful or cause functional deficits depending on the type of nerve involved.^2^^-^5 They most commonly occur in the head and neck involving the brachial plexus and spinal nerves, with the upper and lower extremities being less often affected.^6^

MPNSTs in the general population have a reported incidence of 0.001%; however, in patients with neurofibromatosis, 1 incidence is reported at 8% to 13%. When schwannomas and MPSTs are compared, MPSTs are more commonly found on the limb, are painful, and found deep to the fascia whereas schwannomas are more commonly found in the head, neck, and trunk, are painless, and are superficial to the deep fascia.^7^ Radiographic (magnetic resonance imaging [MRI]) investigations are helpful in finding the origin of the tumor and the relationship of masses with nearby structures. MRI can differentiate malignant versus benign with good sensitivity and specificity; however, it cannot differentiate schwannomas and neurofibromas.^7^ Neurofibromas are common in patients with NF1 and NF2 and are similar to schwannomas in the fact that they both arise from Schwann cells; however, their surgical resection is quite different. Neurofibromas involving a nerve are intimately connected and require resection of the involved nerve segment, whereas schwannomas are well encapsulated and can be removed without disruption of the nerve.^1^

Diagnosis is typically made by history, physical examination, radiographic investigation, and final pathology after incisional versus excisional biopsy. Schwannomas are encapsulated and can be completely enucleated during surgery. Surgical excision is the most effective method of therapy while preserving the nerve function.^8^ Meticulous dissection with magnification can achieve complete tumor removal without neurological loss or recurrence.^2^ Intracapsular tumor removal provides good results with a low complication rate.^2^ If nerve fascicles are damaged during enucleation, the nerve gaps can be reconstructed with nerve grafts to preserve the nerve function.^7^ Paresthesia is the most common postoperative complication.^8^ Malignant transformation and relapse after incision are rare.^8^ Malignancy is suspected if the lesion does not seem to be encapsulated or to adhere to the surrounding tissue; treatment often requires transection of the nerve for removal with repair.^6^

Clinical diagnosis of a schwannoma is not always straightforward due to the large differential diagnosis. Radiographic investigation such as MRI may be helpful in establishing a correct diagnosis. Treatment is achieved by enucleation with preservation of nerve function.

## Figures and Tables

**Figure 1:**
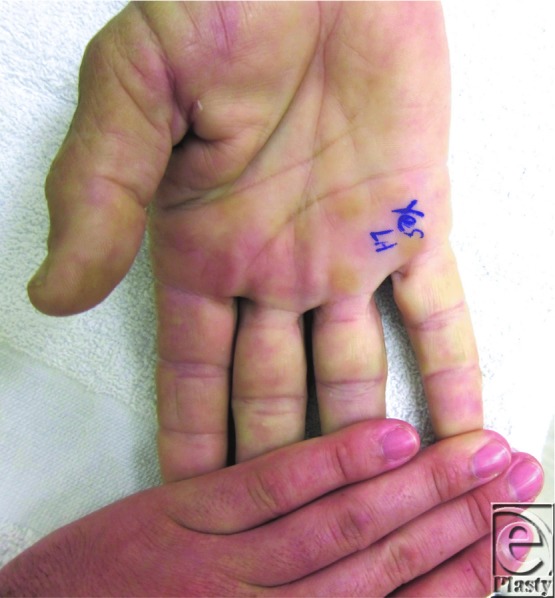
Preoperative right hand: 0.8-cm mass on radiovolar aspect of SF P1, restricted movement in axial direction, freely movable in volar/dorsal direction, negative Tine, nonpulsatile, normal sensation. Because of the location, there was some concern about involvement of the neurovascular bundle. Radiographs were obtained with no foreign visible body.

**Figure 2:**
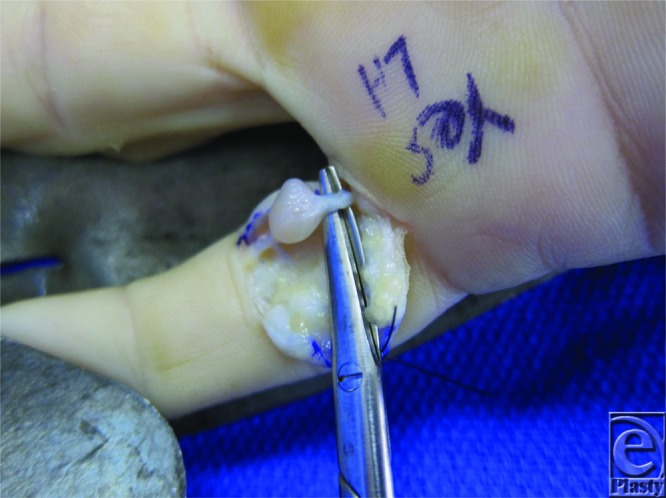
Operative view: 7 × 7-mm firm mass adherent to the proximal small finger digital radial nerve. Mass was well encapsulated and enucleated without damage to the digital nerve.
